# Editorial for “Impact of Regorafenib on Endothelial Transdifferentiation of Glioblastoma Stem-like Cells”

**DOI:** 10.3390/cancers15153830

**Published:** 2023-07-28

**Authors:** Madhukrishna Kolothara Unnikrishnan, Mirko H. H. Schmidt

**Affiliations:** Institute of Anatomy, Medical Faculty Carl Gustav Carus, Technische Universität Dresden School of Medicine, Fetscherstr 74, 01307 Dresden, Germany; madhukrishna.kolothara_unnikrishnan@tu-dresden.de

Glioblastoma multiforme (GBM) is the most frequently occurring form of malignant primary brain tumor in adults [1]. This highly aggressive tumor has a poor prognosis with a significantly short median survival of 12–15 months. Current treatments, including surgery, radiation, and chemotherapy, have limited success in improving survival outcomes [2,3]. In recent treatment developments, GBM has been established as a highly heterogeneous malignancy, increasing the complexity of treatment and difficulty of predicting the outcomes of different treatment paradigms [4]. For example, recurrences of GBM can occur in areas treated with radiation, indicating radioresistance. Additionally, GBM has demonstrated resistance to chemotherapeutic drugs, including temozolomide [5,6,7].

Notably, the presence of self-renewing, pluripotent GBM stem-like cells (GSCs) is believed to allow GBM microtumors to persist after surgery or radiation and differentiate into various tumor-supporting cell types, likely contributing to this common recurrence [8]. For example, tumor-derived endothelial cells (TDECs), formed via the transdifferentiation of GSCs, play an essential role in angiogenesis, a fundamental process that supports tumor proliferation [9,10]. Despite significant progress in elucidating the molecular pathways that underlie GBM pathology, improvements in clinical treatment are still lagging behind. Targeted therapy aimed at intrinsically altered molecules and pathways represents a promising course of action in GBM treatment. Classes of molecules, such as tyrosine kinases (TKs), have been extensively studied, as they are frequently involved in GBM pathology [11]. EGFR, FGFR, PDGFR, VEGFR, and Tie2 and their downstream signaling are significant examples of pathways implicated in various properties of GBM, such as GSC self-renewal, invasion, angiogenesis, and GSC transdifferentiation. Therefore, inhibiting TKs present a potential for targeted treatment for GBM [12,13].

Over the past two decades, tyrosine kinase inhibitors (TKIs) have emerged as a promising treatment for various tumors. TKIs directly or indirectly disrupt TK signaling pathways, inhibiting cell growth, proliferation, angiogenesis, and differentiation [14,15]. In the context of brain tumors, several TKIs have been investigated and have been approved for clinical application. In the case of GBM for example, EGFR inhibitors have demonstrated significant anti-GBM activity, such as tumor growth inhibition and prolonged survival in animal models [12].

Regorafenib is an orally administered TKI that targets a broad spectrum of TKs relevant to GBM pathology. Currently, regorafenib is approved for treating various types of cancers, such as refractory metastatic colorectal cancer and advanced gastrointestinal tumors. In independent preclinical studies and clinical trials, regorafenib has been established as a potential treatment for gastric cancer, sarcomas, and various brain tumors, including GBM [16,17].

Deshors et al., in their research paper entitled “Impact of Regorafenib in Endothelial Transdifferentiation of Glioblastoma Stem-like Cells”, published in the 14th volume of *Cancers*, 2022, addresses the significance of the transdifferentiation of GSCs into TDECs in GBM development. The transdifferentiation of GSCs into TDECs contributes to the abnormal and abundant vascularization of GBM, an essential process in tumor growth [10]. The authors investigated the influence of regorafenib, a promising TKI, in GSC self-renewal, tumorigenicity, and transdifferentiation, providing further insights into the efficacy of regorafenib as a potential treatment for GBM [18].

GSCs are characterized by self-renewal and tumorigenicity. Using surgical GSC samples, Deshors et al. assessed GSC self-renewal capacity via neurosphere formation, a process in which regorafenib induced a significant, dose-dependent reduction. Furthermore, in concentrations above 3 µM, regorafenib demonstrated high toxicity toward GSCs. An orthotopic xenograft nude mouse model was used to evaluate the tumorigenicity of GSCs. The oral administration of regorafenib in mice significantly reduced tumor growth compared to that in untreated mice. Furthermore, immunostaining of the endothelial marker CD31 revealed a significant reduction in CD31-positive vessels in tumors treated with regorafenib compared to tumors in the untreated mice. This significant reduction in overall tumor growth, along with the endothelial characteristics of the xenograft model following regorafenib treatment, led the authors to specifically assess the effect of regorafenib on the transdifferentiation of GSCs into TDECs. Notably, they found that low concentrations of regorafenib (1–2 µM) did not significantly affect GSC viability. Nevertheless, in an assessment that measured AKT phosphorylation as a proxy for TK activity, it was found that regorafenib effectively reduced TK signaling in GSCs. Regorafenib caused a dose-dependent reduction in CD31 protein expression in TDECs. In addition, a reduction in the number of live CD31-positive cells was observed via flow cytometry analysis, further supporting this observation. In a specific assessment of the angiogenic character of TDEC, it was found that regorafenib induced a dose-dependent decrease in pseudotube formation in TDEC.

In addition to GSC radioresistance contributing to GBM recurrence, studies describe another phenomenon in which GSC transdifferentiation is potentiated via irradiation (IR) [19]. The authors previously described the involvement of the Tie2 TK signaling pathway in the IR-induced transdifferentiation of GSCs to TDECs [20]. The authors observed that when GSCs were irradiated, the resulting TDECs (TDEC IR+) were already sensitive to regorafenib at a 1–2 µM concentration in a dose-dependent manner compared to the untreated controls. Furthermore, the number of CD31-positive live endothelial cells significantly decreased in the TDEC IR+ following regorafenib treatment. CD31 expression as well as TDEC pseudotube formation were also significantly reduced in response to regorafenib.

The authors previously demonstrated that IR-induced transdifferentiation is partly driven by the activation of Tie2 signaling [20]. No significant change in Tie2 expression was observed in TDEC IR+ at 1 µM regorafenib, while 2 µM significantly reduced Tie2 expression and its phosphorylation. Notably, regorafenib did not reduce Tie2 activation in TDEC IR- and only a high dose triggered a reduction in Tie2 expression in GSC. Previous studies found that the Tie2 signaling pathway is involved in transdifferentiation but is not essential. However, this pathway plays a crucial role in IR-induced transdifferentiation. Taken together, provided it is not excessively toxic to cell proliferation, regorafenib at a high dose inhibits the IR-induced transdifferentiation of GSCs by inhibiting the Tie2 pathway.

Using the Matrigel plug assay, the authors evaluated the effect of regorafenib on classical (IR-) and IR-induced (IR+) transdifferentiation in vivo. Subcutaneous implantation of either TDEC IR+ or TDEC IR- was made along with the oral administration of regorafenib or a control. The authors observed that plugs with TDEC IR- treated with regorafenib contained significantly fewer functional blood vessels than TDEC IR- treated with the control. Additionally, plugs containing TDEC IR+ control contained significantly more functional blood vessels than the TDEC IR- control, corroborating previous findings that irradiation potentiates transdifferentiation [20]. Remarkably, the TDEC IR+ plug treated with regorafenib contained significantly fewer functional blood vessels than TDEC IR+ treated with the control. Crucially, these findings suggest that regorafenib inhibited both classical and IR-induced GSC transdifferentiation in vivo.

Despite aggressive treatment combining resection, radiation, and chemotherapy, GBM remains a formidable disease, with no significant improvement in survival having been made over the last three decades. A great number of studies highlight TKIs as promising drugs against GBM, and independent studies show regorafenib to be a promising choice in treating recurrent GBM when other drugs prove unsuccessful [21]. Deshors et al. shed light on the influence of regorafenib on essential aspects of GBM development, demonstrating for the first time that this TKI inhibits GSCs as well as the transdifferentiation of GSCs to TDEC.

There has been a clear need to find vulnerabilities in the molecular pathology of GBM to develop targeted therapies. There is growing evidence that TKIs have great potential for treating GBM. However, TKI-based treatment also faces challenges, such as tumor heterogeneity, dysregulated molecular pathways, and, most notably, the presence of the blood–brain barrier (BBB). The ability to cross the BBB is a crucial determinant of drug efficacy in brain tumors [12,22]. Clinical studies on BBB permeability observed a sufficient concentration of regorafenib in the cerebrospinal fluid. However, regorafenib’s intrinsic properties decrease the availability of free drug that can induce pharmacological effects [23,24]. Despite these challenges, there are various methods to enhance the efficiency of drug delivery. For example, altering BBB permeability, bypassing BBB through alternate routes such as intranasal delivery or direct injection, and nanoparticles are all promising strategies and can be applied in combination [12]. The development of potent anti-tumor TKIs, such as regorafenib, in combination with effective drug delivery methods, could encourage significant progress toward treating GBM.

## References

[1] Furnari F.B., Fenton T., Bachoo R.M., Mukasa A., Stommel J.M., Stegh A., Hahn W.C., Ligon K.L., Louis D.N., Brennan C. (2007). Malignant Astrocytic Glioma: Genetics, Biology, and Paths to Treatment. Genes Dev..

[2] Stupp R., Mason W.P., Van Den Bent M.J., Weller M., Fisher B., Taphoorn M.J.B., Belanger K., Brandes A.A., Marosi C., Bogdahn U. (2005). Radiotherapy plus Concomitant and Adjuvant Temozolomide for Glioblastoma. N. Engl. J. Med..

[3] Ostrom Q.T., Patil N., Cioffi G., Waite K., Kruchko C., Barnholtz-Sloan J.S. (2020). CBTRUS Statistical Report: Primary Brain and Other Central Nervous System Tumors Diagnosed in the United States in 2013–2017. Neuro-Oncology.

[4] Tang D.G. (2012). Understanding Cancer Stem Cell Heterogeneity and Plasticity. Cell Res..

[5] Seymour T., Nowak A., Kakulas F. (2015). Targeting Aggressive Cancer Stem Cells in Glioblastoma. Front. Oncol..

[6] Arora A., Somasundaram K. (2019). Glioblastoma vs. Temozolomide: Can the Red Queen Race Be Won?. Cancer Biol. Ther..

[7] Alves A.L.V., Gomes I.N.F., Carloni A.C., Rosa M.N., Da Silva L.S., Evangelista A.F., Reis R.M., Silva V.A.O. (2021). Role of Glioblastoma Stem Cells in Cancer Therapeutic Resistance: A Perspective on Antineoplastic Agents from Natural Sources and Chemical Derivatives. Stem Cell Res. Ther..

[8] Hardee M.E., Zagzag D. (2012). Mechanisms of Glioma-Associated Neovascularization. Am. J. Pathol..

[9] Scully S., Francescone R., Faibish M., Bentley B., Taylor S.L., Oh D., Schapiro R., Moral L., Yan W., Shao R. (2012). Transdifferentiation of Glioblastoma Stem-like Cells into Mural Cells Drives Vasculogenic Mimicry in Glioblastomas. J. Neurosci. Off. J. Soc. Neurosci..

[10] Ricci-Vitiani L., Pallini R., Biffoni M., Todaro M., Invernici G., Cenci T., Maira G., Parati E.A., Stassi G., Larocca L.M. (2010). Tumour Vascularization via Endothelial Differentiation of Glioblastoma Stem-like Cells. Nature.

[11] El Atat O., Naser R., Abdelkhalek M., Habib R., El Sibai M. (2022). Molecular Targeted Therapy: A New Avenue in Glioblastoma Treatment (Review). Oncol. Lett..

[12] Brar H.K., Jose J., Wu Z., Sharma M. (2022). Tyrosine Kinase Inhibitors for Glioblastoma Multiforme: Challenges and Opportunities for Drug Delivery. Pharmaceutics.

[13] Tilak M., Holborn J., New L.A., Lalonde J., Jones N. (2021). Receptor Tyrosine Kinase Signaling and Targeting in Glioblastoma Multiforme. Int. J. Mol. Sci..

[14] Druker B.J., Talpaz M., Resta D.J., Peng B., Buchdunger E., Ford J.M., Lydon N.B., Kantarjian H., Capdeville R., Ohno-Jones S. (2001). Efficacy and Safety of a Specific Inhibitor of the BCR-ABL Tyrosine Kinase in Chronic Myeloid Leukemia. N. Engl. J. Med..

[15] Huang L., Jiang S., Shi Y. (2020). Tyrosine Kinase Inhibitors for Solid Tumors in the Past 20 Years (2001–2020). J. Hematol. Oncol..

[16] Grothey A., Blay J.-Y., Pavlakis N., Yoshino T., Bruix J. (2020). Evolving Role of Regorafenib for the Treatment of Advanced Cancers. Cancer Treat. Rev..

[17] Daudigeos-Dubus E., Le Dret L., Lanvers-Kaminsky C., Bawa O., Opolon P., Vievard A., Villa I., Pagès M., Bosq J., Vassal G. (2015). Regorafenib: Antitumor Activity upon Mono and Combination Therapy in Preclinical Pediatric Malignancy Models. PLoS ONE.

[18] Deshors P., Arnauduc F., Boëlle B., Cohen-Jonathan Moyal E., Courtade-Saïdi M., Evrard S.M. (2022). Impact of Regorafenib on Endothelial Transdifferentiation of Glioblastoma Stem-like Cells. Cancers.

[19] De Pascalis I., Morgante L., Pacioni S., D’Alessandris Q.G., Giannetti S., Martini M., Ricci-Vitiani L., Malinverno M., Dejana E., Larocca L.M. (2018). Endothelial Trans-Differentiation in Glioblastoma Recurring after Radiotherapy. Mod. Pathol..

[20] Deshors P., Toulas C., Arnauduc F., Malric L., Siegfried A., Nicaise Y., Lemarié A., Larrieu D., Tosolini M., Cohen-Jonathan Moyal E. (2019). Ionizing Radiation Induces Endothelial Transdifferentiation of Glioblastoma Stem-like Cells through the Tie2 Signaling Pathway. Cell Death Dis..

[21] Lombardi G., De Salvo G.L., Brandes A.A., Eoli M., Rudà R., Faedi M., Lolli I., Pace A., Daniele B., Pasqualetti F. (2019). Regorafenib Compared with Lomustine in Patients with Relapsed Glioblastoma (REGOMA): A Multicentre, Open-Label, Randomised, Controlled, Phase 2 Trial. Lancet Oncol..

[22] Da Ros M., De Gregorio V., Iorio A., Giunti L., Guidi M., De Martino M., Genitori L., Sardi I. (2018). Glioblastoma Chemoresistance: The Double Play by Microenvironment and Blood-Brain Barrier. Int. J. Mol. Sci..

[23] Zeiner P.S., Kinzig M., Divé I., Maurer G.D., Filipski F., Harter P.N., Senft C., Bähr O., Hattingen E., Steinbach J.P. (2019). Regorafenib CSF Penetration, Efficacy, and MRI Patterns in Recurrent Malignant Glioma Patients. J. Clin. Med..

[24] Guntner A.S., Peyrl A., Mayr L., Englinger B., Berger W., Slavc I., Buchberger W., Gojo J. (2020). Cerebrospinal Fluid Penetration of Targeted Therapeutics in Pediatric Brain Tumor Patients. Acta Neuropathol. Commun..

